# Association of Changes in Creatinine and Potassium Levels After Initiation of Renin Angiotensin Aldosterone System Inhibitors With Emergency Department Visits, Hospitalizations, and Mortality in Individuals With Chronic Kidney Disease

**DOI:** 10.1001/jamanetworkopen.2018.3874

**Published:** 2018-11-02

**Authors:** Katherine G. Garlo, David W. Bates, Diane L. Seger, Julie M. Fiskio, David M. Charytan

**Affiliations:** 1 Department of Medicine, Renal Division, Brigham & Women’s Hospital, Boston, Massachusetts; 2 The Center for Patient Safety Research and Practice, Division of General Internal Medicine and Primary Care, Brigham & Women’s Hospital, Boston, Massachusetts; 3 Clinical and Quality Analysis, Partners HealthCare, Somerville, Massachusetts

## Abstract

**Importance:**

Renin angiotensin aldosterone system inhibitors (RAASIs) benefit individuals with chronic kidney disease (CKD). Elevations in serum creatinine and potassium levels are common reasons for discontinuation of this therapy, but their incidence and risks are not well characterized in community practice.

**Objective:**

To evaluate associations of increased creatinine levels, hyperkalemia, and therapy continuation with the risk of emergency department (ED) visits, hospitalizations, and mortality within 1 year after RAASI therapy initiation in individuals with CKD.

**Design, Setting, and Participants:**

This prospective cohort study included 4661 individuals with nondialysis CKD newly prescribed a RAASI or a diuretic who were treated at 36 outpatient primary care offices affiliated with Brigham & Women’s Hospital and Massachusetts General Hospital, Boston, from January 1, 2009, through December 31, 2011. Individuals receiving a new prescription for a diuretic were used to provide context. All participants had a baseline measure of renal function and at least 1 follow-up measurement of creatinine and potassium levels within 90 days of the prescription. Data were analyzed from January 1, 2009, through December 31, 2012.

**Exposures:**

Changes in creatinine and potassium levels within 90 days after the prescription date and therapy discontinuation.

**Main Outcomes and Measures:**

Emergency department visits, hospitalizations, and mortality within 1 year.

**Results:**

A total of 4661 individuals were included in the analysis (2506 [53.8%] women; mean [SD] age, 71 [14]; 3931 [84.3%] white; and 4198 [90.1%] with CKD stage 3). Of these, 2354 individuals (50.5%) received RAASIs and 2307 (49.5%) received diuretics. Creatinine level increase of at least 30% after RAASI therapy initiation was found in 158 of 2354 individuals (6.7%); hyperkalemia of greater than 5.0 mEq/L, in 251 of 2354 (10.7%). Increases in creatinine level of at least 30% (unadjusted odds ratio [OR], 1.40; 95% CI, 0.89-2.21), hyperkalemia (unadjusted OR, 1.15; 95% CI, 0.64-2.06), and therapy discontinuation (unadjusted OR, 1.01; 95% CI, 0.71-1.46) were not associated with ED visits or hospitalizations, which was consistent with results from competing risk analyses. Initial increases in creatinine level of at least 30% were associated with mortality in the total cohort (adjusted OR [aOR], 2.17; 95% CI, 1.45-3.25). However, the effect was only independent for diuretics (aOR, 2.27; 95% CI, 1.41-3.66) and not for RAASIs (aOR, 1.82; 95% CI, 0.83-3.99).

**Conclusions and Relevance:**

Acute creatinine and potassium level disturbances after initiation of RAASI therapy in individuals with CKD appear to be sustained often often not sustained and not associated with ED visits or hospitalizations, despite therapy continuation. Findings from this study suggest that increases in creatinine level were independently associated with mortality among individuals prescribed diuretics but not RAASIs. Structured laboratory monitoring during RAASI therapy initiation may guide appropriate continuation of therapy in the outpatient setting.

## Key Points

**Question:**

Are acute increases in creatinine levels and hyperkalemia after initiation of renin angiotensin aldosterone system inhibitor (RAASI) therapy associated with a risk of emergency department visits, hospitalization, or mortality at 1 year in patients with chronic kidney disease?

**Findings:**

In this cohort study of 4661 patients with chronic kidney disease, increases in creatinine level and hyperkalemia after initiation of RAASI therapy were not associated with emergency department visits or hospitalizations and often resolved at a second measurement. Mortality was increased among individuals with an increase in creatinine level of at least 30% but the association was not significant after adjustment.

**Meaning:**

Structured laboratory monitoring may guide appropriate continuation of RAASI therapy for outpatient health care professionals, but closer monitoring may be needed for individuals with acute increases in creatinine levels.

## Teaser

This cohort study evaluated associations of increased creatinine levels, hyperkalemia, and continuation of therapy with the risk of emergency department visits, hospitalizations, and mortality within 1 year after initiation of renin angiotensin aldosterone system inhibitor (RAASI) therapy in patients with chronic kidney disease.

## Introduction

Renin angiotensin aldosterone system inhibitors (RAASIs) are among the most commonly prescribed medications. In randomized clinical trials (RCTs), they reduced blood pressure, delayed chronic kidney disease (CKD),^1,2^ improved cardiovascular outcomes, and decreased mortality^3^; benefits accrue across age,^4^ race,^5^ and comorbidities.^6,7,8,9^ Therefore, RAASIs are considered first-line treatments for hypertension and secondary prevention of cardiovascular events.^10,11,12^

Elevations in serum creatinine and potassium levels are consequences of RAASI therapy. Although RCTs suggest that long-term benefits outweigh these acute risks,^13,14,15,16^ recent studies raise concern for patient harm and increased health care expenditure potentially associated with these events. A nationwide cohort study from the United Kingdom^15^ reported increased mortality, cardiovascular events, and end-stage renal disease with incremental rises in serum creatinine levels after initiation of RAASI therapy. Similarly, among cardiovascular agents in the United States, RAASIs were the most frequently associated with emergency department (ED) use owing to adverse drug events, leading to inpatient hospitalization in as many as 25% of cases.^17^ Individuals with CKD are susceptible to rises in creatinine and potassium levels as well as increased risk of ED visits requiring hospitalizations.^18,19^

Such findings heighten awareness of the potential harms of RAASI therapy in real-world settings. In clinical practice, a rise of at least 30% in creatinine level from baseline after RAASI therapy initiation has been adapted as a threshold for discontinuation of therapy,^13^ although evidence outside RCTs is lacking, and no formal guidelines exist. More than 90% of prescription therapy is initiated in outpatient offices, yet the burden of creatinine and potassium level disturbances after RAASI therapy initiation in community practices is not well described. To investigate the relevance of this threshold, we conducted a prospective cohort study of individuals with CKD who initiated outpatient RAASI therapy from their primary care physicians. The aims of our study were to (1) describe rates of acute changes in creatinine and potassium levels, (2) evaluate associations of changes in creatinine and potassium levels with ED visits and hospitalizations combined and with mortality at 1 year, and (3) describe the effects of medication discontinuation on these outcomes. To contextualize these changes, new diuretic prescriptions were included.

## Methods

### Study Design and Patient Population

We assembled a prospective cohort in outpatient primary care offices affiliated with Partners HealthCare, Boston, Massachusetts, of participants with CKD prescribed a RAASI or a diuretic from January 1, 2009, December 31, 2011, with follow-up extended to December 31, 2012. This network includes 36 primary care practices affiliated with Brigham & Women’s Hospital and Massachusetts General Hospital, Boston, and includes 1718 prescribers.^20,21^ The inclusion criteria were (1) a baseline measure of renal function demonstrating preexisting, predialysis CKD stages 3 to 5, (2) a newly prescribed RAASI or diuretic, and (3) follow-up laboratory monitoring for serum creatinine levels within 90 days after the prescription date. Diuretic use was selected for a comparison group, given their common use in outpatient practices for individuals with CKD and analogous disturbances in serum creatinine and electrolyte levels that generally require close laboratory monitoring. This study follows the Strengthening the Reporting of Observational Studies in Epidemiology (STROBE) reporting guideline. The study was approved by the institutional review board of Partners HealthCare, who waived the need for informed consent.^20,21^

Preexisting CKD was determined from the mean of the 3 most recent estimated glomerular filtration rates (eGFRs) measured at least 90 days apart and no more than 1 year before the prescription date. Estimated GFR was automatically calculated in the electronic medical records system by the Modification of Diet in Renal Disease Study equation^22^ from serum creatinine levels. Individuals with a baseline creatinine level of at least 6 mg/dL (to convert to micromoles per liter, multiply by 88.4) or eGFR of no greater than 10 mL/min/1.73 m^2^ were considered to have end-stage renal disease and were excluded. Standard CKD staging was used (stage 3, eGFR of >30 to 60 mL/min/1.73 m^2^; stage 4, eGFR >15 to 30 mL/min/1.73 m^2^; and stage 5, eGFR ≤15 mL/min/1.73 m^2^).^23^ Baseline serum potassium level was determined from the most recent concentration less than 90 days before the prescription date. For individuals with ED or hospitalization events during follow-up, the most proximate serum potassium concentration less than 90 days before the event was obtained.

New prescriptions were distinguished from existing prescriptions by requiring the absence of any prescription for that class for at least 6 months before the prescription date. The RAASI group included angiotensin-converting enzyme inhibitors and angiotensin receptor blockers. The diuretic group included loop, thiazide, combined β-blocker and thiazide, and combined α-blocker and thiazide. Combinations of RAASIs or diuretics and mineralocorticoid receptor antagonists were excluded to allow individual assessment of RAASI or diuretic effects. Similarly, participants who were initially prescribed a RAASI and later prescribed a diuretic or vice versa at any time during the study period were excluded. Individuals were excluded if the first follow-up laboratory measurement was within 2 days and the next follow-up laboratory measurement was at least 90 days after the prescription date because changes in creatinine and potassium levels that occurred within 2 days were unlikely to be related to the new prescription. Where the first follow-up laboratory measurement occurred within 2 days and the second within 90 days after the prescription date, the second follow-up laboratory measurement was used. Individuals in whom the therapy was discontinued before the first follow-up laboratory measurement were excluded. All descriptive variables were obtained from the electronic medical record with comorbidities captured from the clinical problem list.

### Exposures and Outcomes

The primary laboratory exposures were selected pragmatically from commonly used markers in outpatient follow-up. They included (1) change in serum creatinine level of less than 30% or at least 30% (subcategorized as 30%-49%, 50%-199%, and >200%) between baseline and the first follow-up laboratory measurement within 90 days after the prescription date; (2) hyperkalemia defined as a potassium level of greater than 5.0 mEq/L or as greater than 5.5 mEq/L and hypokalemia defined as less than 3.4 mEq/L (to convert to millimoles per liter, multiply by 1.0) on the first follow-up laboratory measurement within 90 days of prescription date; and (3) therapy discontinuation between the first and second follow-up laboratory measurements and at any time before the ED visit or hospitalization.

The primary end points included (1) event rates and time to first ED visit or hospitalization within 1 year of the first follow-up laboratory measurement and (2) event rates and time to mortality within 1 year of the first follow-up laboratory measurement. The rate of sustained and nonsustained creatinine level changes on the second follow-up laboratory measurement in individuals with a rise in creatinine level of at least 30% from baseline on the first follow-up laboratory measurement was a secondary outcome.

### Statistical Analysis

Data were analyzed from January 1, 2009, through December 31, 2012. Baseline demographics, comorbidities, and laboratory values were compared between groups using unpaired 2-tailed *t* tests for continuous variables, χ^2^ tests for categorical variables, and trend tests for race, insurance, and CKD stage. Differences in the primary outcomes between RAASI and diuretic use were compared with χ^2^ tests, and logistic regression was used to calculate the odds of a sustained rise in creatinine level of at least 30% from baseline on the second follow-up laboratory measurement. Logistic regression was used to test for association with 2 separate outcomes, including ED visit or hospitalization and mortality within 365 days from the first follow-up laboratory measurement. The indicators were initial creatinine level change, sustained creatinine level change, potassium level disturbance, prescription type, and therapy continuation. To better isolate medication effect, only individuals with normal baseline potassium levels (3.4-5.0 mEq/dL) were included in analyses of associations between serum potassium level changes. Associations with time to ED visit or hospitalization were assessed with a competing risk model that included death as a competing risk according to the method of Fine and Gray.^22^ This analysis accounts for a diminishing risk set from censoring owing to mortality and is often used in CKD cohort studies.^24,25^ Three group comparisons were made for creatinine level change of at least 30% vs less than 30%, potassium level disturbances (hyperkalemia, hypokalemia, and normokalemia), and therapy discontinuation vs continuation. All analyses were run in Stata software (version 14.2; StataCorp) using a 2-sided test and *P* < .05 for significance.

## Results

### Study Population

Among 8271 individuals with CKD stages 3 to 5 (eGFR ≤60 mL/min/1.73 m^2^) not undergoing dialysis, 4661 (2155 men [46.2%] and 2506 women [53.8%]; mean [SD] age, 71 [14] years; 3931 [84.3%] white; 4198 [90.1%] CKD stage 3) had a follow-up laboratory evaluation within 90 days of RAASI (2354 [50.5%]) or diuretic (2307 [49.5%]) initiation (Figure and Table 1). Of the 3610 excluded participants, 2861 (79.3%) lacked a laboratory measurement within 90 days from the prescription date (Figure). Baseline characteristics in this group were similar to those of the participants included (eTable 1 in the Supplement). The mean (SD) duration from the therapy start date to the first follow-up laboratory date was 29.3 (24.5) days; the median was 20 days (interquartile range, 8-43 days); and the data were normally distributed. The most common RAASIs were lisinopril (1871 [71.8%]), valsartan (206 [7.9%]), and losartan potassium (159 [6.1%]). Furosemide (1567 [61.0%]), hydrochlorothiazide (672 [26.2%]), and combined triamterene and hydrochlorothiazide (152 [5.9%]) were the most common diuretics (eTable 1 in the Supplement). Individuals prescribed diuretics were more often older (mean [SD] age, 73 [14] vs 70 [14] years; *P* < .001), female (1312 [56.9%] vs 1194 [50.7%]; *P* < .001), white (1961 [85.0%] vs 1970 [83.7%]; *P* = .005), and insured through Medicare or Medicaid (1331 [57.7%] vs 1267 [53.8%]; *P* = .02) compared with those prescribed RAASIs. Comorbidities were also more frequent in the diuretic group, with higher frequencies of diabetes (655 [27.8%] vs 444 [19.2%]; *P* < .001), hypertension (1435 [61.0%] vs 1295 [56.1%]; *P* = .02), cardiovascular disease (467 [19.8%] vs 616 [26.7%]; *P* < .001), and advanced CKD (30 [1.3%] vs 55 [2.4%]; *P* < .001). The diuretic group also had poorer baseline kidney function and higher potassium levels (Table 1). Data were complete for all baseline variables except race and potassium level (missing in 104 [2.2%] and 47 [1.0%], respectively). For full comparisons of included and excluded participants by drug class, see eTable 2 in the Supplement.

**Figure.**
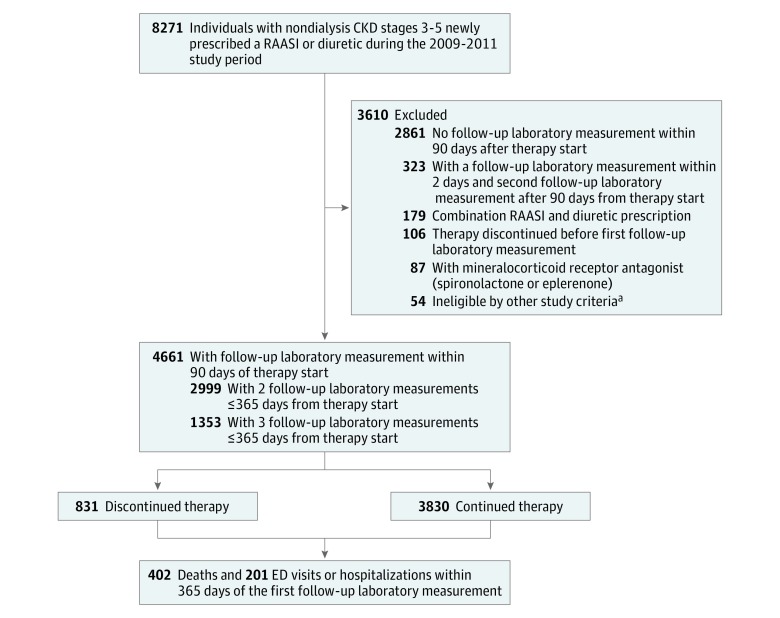
Study Flowchart Stages 3 to 5 chronic kidney disease (CKD) are defined as an estimated glomerular filtration rate (eGFR) of no greater than 60 mL/min/1.73 m^2^. ED indicates emergency department; RAASI, renin angiotensin aldosterone system inhibitor. ^a^Baseline creatinine level of at least 6 mg/dL and eGFR of no greater than 10 mL/min/1.73 m^2^.

**Table 1.** Baseline Characteristics of the Study Population

Characteristic	Treatment Group		
	All (N = 4661)	RAASI (n = 2354)	Diuretic (n = 2307)
Demographic			
Age, mean (SD), y	71 (14)	70 (14)^a^	73 (14)
Age ≥65 y, No. (%)	3276 (70.3)	1572 (66.8)^a^	1704 (73.9)
Male, No. (%)	2155 (46.2)	1160 (49.3)	995 (43.1)
Race, No. (%)^b^			
White	3931 (84.3)	1970 (83.7)^a^	1961 (85.0)
Black	285 (6.1)	141 (6.0)^a^	144 (6.2)
Hispanic	143 (3.1)	85 (3.6)^a^	58 (2.5)
Asian	84 (1.8)	58 (2.5)^a^	26 (1.1)
Other	16 (0.3)	7 (0.3)^a^	9 (0.4)
Insurance, No. (%)			
Medicare or Medicaid	2598 (55.7)	1267 (53.8)^a^	1331 (57.7)
Private	1906 (40.9)	1002 (42.6)^a^	904 (39.2)
MassHealth	75 (1.6)	48 (2.0)^a^	27 (1.2)
Self-pay	82 (1.8)	37 (1.6)^a^	45 (2.0)
Comorbidities and medications, No. (%)			
CKD stage			
3	4198 (90.1)	2172 (92.3)^a^	2026 (87.8)
4	382 (8.2)	152 (6.5)^a^	230 (10.0)
5	81 (1.7)	30 (1.3)^a^	51 (2.2)
Diabetes	1099 (23.6)	655 (27.8)^a^	444 (19.2)
Hypertension	2730 (58.6)	1435 (61.0)^a^	1295 (56.1)
Cardiovascular disease^c^	1083 (23.2)	467 (19.8)^a^	616 (26.7)
Hyperlipidemia	1075 (23.1)	568 (24.1)	507 (22.0)
NSAIDs	856 (18.4)	459 (19.5)^a^	397 (17.2)
Baseline laboratory values, mean (SD)			
Creatinine, mg/dL	1.43 (0.7)	1.39 (0.6)^a^	1.48 (0.9)
eGFR, mL/min/1.73 m^2^	45.7 (10.9)	46.6 (10.2)^a^	44.7 (11.6)
Potassium, mEq/L^d^	4.3 (0.5)	4.3 (0.5)^a^	4.2 (0.5)

Abbreviations: CKD, chronic kidney disease; eGFR, estimated glomerular filtration rate; NSAIDs, nonsteroidal anti-inflammatory drugs; RAASI, renin angiotensin aldosterone system inhibitor.

SI conversion factors: To convert creatinine to micromoles per liter, multiply by 88.4; potassium to millimoles per liter, multiply by 1.0.

^a^ *P* < .05 for comparison of RAASI and diuretic groups.

^b^ Race was declined or not given by 104 (2.2%) for all participants, 52 (2.2%) for the RAASI group, and 46 (2.0%) for the diuretics group. Race was missing for 98 (2.1%) of all participants, 41 (1.7%) in the RAASI group, and 63 (2.7%) in the diuretics group.

^c^ Includes preserved and reduced ejection fraction heart failure, coronary artery disease, and valvular disease.

^d^ Baseline potassium level was available in 4614 (99.0%) of all participants, 2328 (98.9%) in the RAASI group, and 2286 (99.1%) of the diuretics group.

### Initial Creatinine and Potassium Level Changes by Threshold Levels on First Follow-up

Creatinine level increases of at least 30% from baseline occurred in 158 of 2354 individuals (6.7%) on the first follow-up laboratory measurement in participants prescribed RAASIs. Preservation of baseline creatinine level (<30% increase) was more frequent in less advanced CKD (2298 of 4198 [54.7%] vs 241 of 463 [52.1%]; *P* = .002) (Table 2). Among individuals with normal baseline potassium level, hyperkalemia (≥5.0 mEq/L) occurred in 251 of 2354 (10.7%) and hypokalemia (<3.4 mEq/L) in 64 of 2354 (2.7%) on the first follow-up laboratory measurement. Hyperkalemia was more common in CKD stages 4 to 5 compared with stage 3 (96 of 463 [20.7%] vs 317 of 4198 [7.6%]; *P* < .001). As expected, differences between the RAASI and diuretic groups for hyperkalemia (251 [10.7%] vs 162 [7.0%]; *P* < .001) and hypokalemia (64 [2.7%] vs 196 [8.5%]; *P* < .001) (Table 2).

**Table 2.** Event Rates for Change in Creatinine and Potassium Levels From Baseline to First Follow-up Laboratory Measurement Within 90 Days of Initiating Therapy by Medication Class and CKD Stage

Event^a^	No. (%) of Participants				
	Total (N = 4661)	Treatment Group		CKD Severity	
		RAASI (n = 2354 [50.5%])	Diuretic (n = 2307 [49.5%])	Stage 3 (n = 4198 [90.1%])	Stages 4-5 (n = 463 [9.9%])
**Serum Creatinine Level**					
Change <30%^a^	4296 (92.2)	2196 (93.3)	2100 (91.0)	3882 (92.5)	414 (89.4)
Decreased	1757 (37.7)	924 (39.2)	833 (36.1)	1584 (37.7)	173 (37.4)
Unchanged	2539 (54.5)	1272 (54.0)	1267 (54.9)	2298 (54.7)	241 52.1)
Change ≥30%	365 (7.8)	158 (6.7)	207 (9.0)	316 (7.5)	49 (10.6)
≥30 to 50 mg/dL	234 (5.0)	92 (3.9)	142 (6.2)	208 (5.0)	26 (5.6)
>50 to 200 mg/dL	125 (2.7)	62 (2.6)	63 (2.7)	105 (2.5)	20 (4.3)
>200 mg/dL	6 (0.1)	4 (0.2)	2 (0.1)	3 (0.1)	3 (0.6)
**Serum Potassium Level**^b^					
Hyperkalemia					
>5.0 mEq/L	413 (8.9)	251 (10.7)^c^	162 (7.0)	317 (7.6)	96 (20.7)
>5.0 to 5.5 mEq/L	323 (6.9)	210 (8.9)^c^	113 (4.9)	269 (6.4)	54 (11.7)
>5.5 to 6.0 mEq/L	71 (1.5)	33 (1.4)^c^	38 (1.6)	38 (0.9)	33 (7.1)
>6.0 mEq/L	19 (0.4)	8 (0.3)^c^	11 (0.5)	10 (0.2)	9 (1.9)
Hypokalemia					
<3.4 mEq/L	260 (5.6)	64 (2.7)^c^	196 (8.5)	245 (5.8)	15 (3.2)
<3.4 to >3.0 mEq/L	211 (4.5)	58 (2.5)^c^	153 (6.6)	200 (4.8)	11 (2.4)
≤3.0 mEq/L	49 (1.1)	6 (0.2)^c^	43 (1.9)	45 (1.1)	4 (0.9)
No change, 3.4 to 5.0 mEq/L	3954 (84.8)	2019 (85.8)	1935 (83.9)	3604 (85.9)	350 (75.6)

Abbreviations: CKD, chronic kidney disease; RAASI, renin angiotensin aldosterone system inhibitor.

SI conversion factors: To convert creatinine to micromoles per liter, multiply by 88.4; potassium to millimoles per liter, multiply by 1.0.

^a^ *P* = .002, χ^2^ test for RAASI vs diuretic.

^b^ Thirty-four patients were missing follow-up information for potassium level, including 20 in the RAASI group, 14 in the diuretic group, 32 with CKD stage 3, and 2 with CKD stages 4 to 5.

^c^ *P* < .001 vs diuretic group, χ^2^ test.

### Associations With ED Visits, Hospitalizations, and Mortality

The 1-year incidence of ED visits and hospitalizations combined for creatinine level change of less than 30% and at least 30% were 4.4% (188 of 4296) and 6.0% (22 of 365), respectively. One-year mortality was 6.3% (271 of 4296) and 11.5% (42 of 365) for creatinine level changes of less than 30% and at least 30%, respectively (eTable 4 in the Supplement). No associations of ED visits or hospitalizations with creatinine level change of at least 30%, potassium level disturbance, medication class, or therapy continuation occurred in unadjusted or adjusted analyses (Table 3). Increases in creatinine level of at least 30% (unadjusted odds ratio [OR], 1.40; 95% CI, 0.89-2.21), hyperkalemia (unadjusted OR, 1.15; 95% CI, 0.64-2.06), and therapy medication discontinuation (unadjusted OR, 1.01; 95% CI, 0.71-1.46) were not associated with ED visits or hospitalizations, which was consistent with results from competing risk analyses. The mean (SD) of the most recent potassium concentrations within 90 days before the ED visit or hospitalization was 4.4 (0.6) mEq/L for individuals with hyperkalemia and 4.1 (0.7) mEq/L for individuals with hypokalemia.

**Table 3.** Associations for ED Visit or Hospitalization and Mortality Within 1 Year of the First Follow-up Laboratory Measurement After RAASI or Diuretic Prescription^a^

Outcome	ED Visit and Hospitalization				Mortality			
	Univariate OR (95% CI)	*P* Value	Multivariate OR (95% CI)	*P* Value	Univariate OR (95% CI)	*P* Value	Multivariate OR (95% CI)	*P* Value
Creatinine level change, %^b^								
<30	1 [Reference]	NA	1 [Reference]	NA	1 [Reference]	NA	1 [Reference]	NA
≥30	1.40 (0.89-2.21)	.15	0.96 (0.62-1.50)	.87	1.87 (1.37-2.56)	<.001	2.17 (1.45-3.25)	<.001
Sustained creatinine level change, %^c^								
<30	1 [Reference]	NA	1 [Reference]	NA	1 [Reference]	NA	1 [Reference]	NA
≥30	0.63 (0.25-1.62)	.34	0.23 (0.04-1.18)	.08	0.61 (0.29-1.27)	.19	0.41 (0.14-1.21)	.12
Potassium level change, mEq/L^d^								
3.4-5.0	1 [Reference]	NA	1 [Reference]	NA	1 [Reference]	NA	1 [Reference]	NA
>5.0	1.15 (0.64-2.06)	.64	1.40 (0.87-2.25)	.16	1.07 (0.68-1.66)	.78	1.07 (0.59-1.92)	.83
<3.4	0.94 (0.46-1.95)	.87	1.20 (0.69-2.09)	.53	2.17 (1.46-3.23)	<.001	1.77 (0.97-3.25)	.07
Medication								
Diuretic	1 [Reference]	NA	1 [Reference]	NA	1 [Reference]	NA	1 [Reference]	NA
RAASI	0.83 (0.63-1.10)	.20	0.94 (0.73-1.20)	.60	0.36 (0.29-0.46)	<.001	0.45 (0.33-0.61)	<.001
Therapy								
Continued	1 [Reference]	NA	1 [Reference]	NA	1 [Reference]	NA	1 [Reference]	NA
Discontinued	1.01 (0.71-1.46)	.92	0.83 (0.47-1.47)	.52	1.08 (0.83-1.41)	.56	1.04 (0.47-2.28)	.93

Abbreviations: ED, emergency department; NA, not applicable; OR, odds ratio; RAASI, renin angiotensin aldosterone system inhibitor.

SI conversion factor: To convert potassium to millimoles per liter, multiply by 1.0.

^a^ Multivariate models are adjusted for age, sex, race, chronic kidney disease stage, cardiovascular disease, hypertension, diabetes, baseline glomerular filtration rate, and baseline potassium level.

^b^ Defined from baseline on first follow-up laboratory measurement within 90 days of the prescription date.

^c^ Defined from baseline on the first and second follow-up laboratory measurement. The second follow-up laboratory measurement was within 365 days of prescription date.

^d^ All individuals had baseline serum potassium levels of 3.4 to 5.0 mEq/L (reference range) and a follow-up potassium level within 90 days of the prescription date.

Creatinine level changes of at least 30% compared with less than 30% were associated with mortality in adjusted analyses (adjusted odds ratio [aOR], 2.17; 95% CI, 1.45-3.25) (Table 3). When analyzed according to medication class, this association remained independent in the diuretic group (aOR, 2.27; 95% CI, 1.41-3.66) but not the RAASI group (aOR, 1.82; 95% CI, 0.83-3.99) (Table 4). The number of individuals with therapy discontinuation and clinical outcomes was small (38 ED visits or hospitalizations and 45 deaths) (Table 3).

**Table 4.** Associations With Mortality Within 1 Year of New RAASI or Diuretic Prescription by Medication Class

Medication Class	Univariate OR (95% CI)	*P* Value	Multivariate OR (95% CI)^a^	*P* Value
Diuretic only				
Creatinine increase ≥30% of baseline^b^	1.74 (1.20-2.53)	.004	2.27 (1.41-3.66)	.001
Sustained creatinine increase ≥30%^c^	0.67 (0.27-1.67)	.40	0.43 (0.92-1.01)	.24
Hypokalemia <3.5 mEq/L^d^	1.61 (1.04- 2.49)	.03	1.73 (0.91-3.26)	.09
Therapy discontinued^e^	1.10 (0.81-1.50)	.54	1.56 (0.55-4.39)	.40
CKD stages 4-5 vs 3	1.00 (0.69-1.47)	.98	0.57 (0.25-1.25)	.16
RAASI only				
Creatinine increase ≥30% of baseline^b^	1.82 (1.0-3.320)	.05	1.82 (0.83-3.99)	.14
Sustained creatinine increase ≥30%^c^	0.58 (0.16-2.13)	.42	0.19 (0.02-1.82)	.15
Hyperkalemia >5 mEq/L^d^	1.22 (0.60- 2.49)	.57	0.78 (0.27-2.27)	.65
Therapy discontinued^e^	0.91 (0.54-1.52)	.72	0.56 (0.16-1.92)	.36
CKD stages 4-5 vs 3	0.76 (0.35-1.65)	.48	0.36 (0.09-1.49)	.16

Abbreviations: CKD, chronic kidney disease; OR, odds ratio; RAASI, renin angiotensin aldosterone system inhibitor.

SI conversion factor: To convert potassium to millimoles per liter, multiply by 1.0.

^a^ Adjusted for the following covariates: age, sex, race, CKD stage, cardiovascular disease, hypertension, diabetes, baseline glomerular filtration rate, baseline potassium level, and medication discontinuation before death.

^b^ Defined by the first follow-up laboratory measurement within 90 days of the prescription date.

^c^ Defined as creatinine level of at least 30% of baseline on the first and second laboratory testing. The second follow-up laboratory measurement was within 365 days of prescription date.

^d^ All individuals had baseline serum potassium levels of 3.5 to 5.0 mEq/L (reference range) before therapy initiation and a follow-up potassium level within 90 days of therapy start.

^e^ Forty participants from the entire cohort had their RAASI or diuretic therapy stopped before death.

### Time to First ED Visit or Hospitalization Within 365 Days After RAASI Therapy Initiation

In risk analyses with death as the competing event, the cumulative incidence of ED visit or hospitalization among individuals prescribed RAASIs did not differ between those with vs without a creatinine level change of at least 30% (subdistribution hazard ratio, 1.02; 95% CI, 0.45-2.32). Neither hyperkalemia nor therapy continuation were indicators of ED visits or hospitalization in the competing risk analyses (eTable 5 in the Supplement).

### Sustained Creatinine Level Changes and Associations With Therapy Discontinuation

The number of participants with an initial increase in serum creatinine level of at least 30% who had second and third follow-up laboratory measurements were small, (249 and 41, respectively) (eTable 3 in the Supplement). More than half (146 [58.6%]) returned to baseline values on the second follow-up measurement and 103 (41.4%) had a sustained increase in levels that persisted in 14 (34.1%) at the third laboratory follow-up measurement (eTable 3 in the Supplement). Two hundred twenty-six individuals (90.8%) with an initial increase in creatinine level of at least 30% from baseline on the first follow-up measurement had their therapy discontinued before the second follow-up laboratory measurement. Although numerically higher, the incidence of sustained rise in creatinine levels of at least 30% was not statistically different between those who continued compared with those who discontinued therapy (OR, 1.96; 95% CI, 0.83-4.67; *P* = .13) (eTable 3 in the Supplement). All discontinuations occurred between the first and second follow-up laboratory measurement; none occurred between the second and third laboratory measurements.

## Discussion

In this cohort of individuals with CKD who initiated RAASI or diuretic therapy in primary care practices, acute elevations in creatinine level of at least 30% occurred in 365 individuals (7.8%) and were more common in those with stages 4 to 5 compared with stage 3 CKD. Increases in creatinine levels of at least 30% were often not sustained on the next follow-up laboratory measurement. Similarly, hyperkalemia occurred in 413 of 4461 individuals (8.9%) and was more common in those with CKD stages 4 to 5 compared with stage 3 CKD. Acute increases in creatinine levels of at least 30% and hyperkalemia were not associated with increased risk of ED visits or hospitalizations. However, in individuals who initiated diuretic therapy, acute elevations in creatinine levels were independently associated with a greater than 2-fold increased risk of mortality. The risks of ED visits, hospitalizations, or mortality did not differ between individuals with therapy continuation compared with those with therapy discontinuation after an initial rise of at least 30% in creatinine level.

Pooled results of RCTs from high-risk populations with CVD^26^ report an initial rise in serum creatinine levels of at least 30% within 2 weeks in 16%, which persist in only 7% at 3 months. Conversely, a quantitative review of 4 RCTs representative of a general-risk population^27^ reported worsening kidney function in only 0.5% to 3.0% of participants at 25 months. Differences between our findings and those of prior studies may be explained by several factors, including differences in the underlying comorbidities of the study populations, starting dose of the prescription, and timing of laboratory follow-up. Our cohort contained a relatively low proportion of individuals with diabetes (1099 [23.6%]) and cardiovascular disease (1083 [23.2%]). Thus, these individuals may have had better renal reserve with less susceptibility to changes in creatinine level from the hemodynamic stress of RAASI therapy compared with higher-risk populations studied.^26^ This interpretation is consistent with our finding of an increased incidence of these events in those with advanced CKD. In addition, the medication dose was likely higher in RCTs because outpatient health care professionals may prescribe lower starting doses with the intention to titrate up if tolerated. Last, we used a 90-day period to the first follow-up laboratory measurement, whereas the time to the first follow-up laboratory measurement was shorter in RCTs, often within 14 days. If creatinine levels peak within the first 2 weeks after initiation of RAASI therapy and then frequently return toward baseline, our methods may have failed to detect these variations. However, if these early changes are transient and self-limited, as suggested by our data and that of others,^13^ using a longer window may provide data on more physiologically important variations in blood chemistry findings. In addition, the extended 90-day window allowed for outpatient prescription fulfilment and assessment of the pattern of changes in a laboratory follow-up in a time frame more consistent with outpatient primary care practice than looking at data gathered solely within 1 to 2 weeks of medication initiation.

Our study is one of few to evaluate whether changes in creatinine levels after RAASI therapy initiation as prescribed in community practices are associated with mortality. A large population-based study from the United Kingdom of 122 363 participants newly prescribed RAASIs during the period from 1997 through 2014 reported positive associations between a rise of creatinine levels of at least 30% and risks of end-stage renal disease, myocardial infarction, heart failure, and mortality when compared with participants without a rise in creatinine levels.^15^ These findings were independent of comorbidities and consistent when stratified by stage of CKD. However, kidney function was defined by a single baseline serum creatinine level within 12 months and single follow-up creatinine level within 2 months of RAASI therapy initiation.^15^ This definition may not provide adequate assessment of baseline or postprescription changes in kidney function, given the potential for transient fluctuations in renal function as shown in our analyses. In addition, only 1.7% developed a rise in creatinine levels of at least 30% compared with 6.7% in our cohort, suggesting that a higher proportion in that study had normal kidney function at baseline. In the United States, RAASIs were reported as the most frequent cardiovascular agent to be associated with ED visits for adverse drug events,^17^ accounting for 3.5% of the national total ED visits for adverse drug events from 2013 to 2014. As many as 25% of these ED visits led to hospitalization.^17^ However, that study was unable to assess whether ED visits were related to the metabolic abnormalities of RAASI therapy initiation. Our analyses expand on these prior studies by reporting no association in ED visits, hospitalizations, or mortality with increases in creatinine levels of at least 30% or hyperkalemia after initiation of a new RAASI prescription. In contrast, changes in serum creatinine levels after initiation of diuretic therapy appeared to be more strongly associated with clinical outcomes. Furthermore, therapy continuation compared with treatment discontinuation did not appear to affect the subsequent risk of clinical events, although this finding is limited by the low numbers of individuals with discontinuation. Similar to our findings, another study^28^ reported no difference in continuation compared with temporary discontinuation of RAASI therapy before coronary angiography and cardiac surgery for the risk of acute kidney injury in hospitalized individuals (relative risk, 1.48; 95% CI, 0.84-2.60).

Our findings suggest that structured laboratory monitoring during RAASI and diuretic therapy initiation may be used to guide appropriate continuation of therapy in individuals with initial changes in kidney function. The acute changes in creatinine levels often resolved on follow-up testing and were not associated with near-term risks of combined ED visit and hospitalization or mortality. Given this finding, the benefits of RAASI therapy in blood pressure control, proteinuria reduction, and long-term preservation of kidney function could outweigh the risk of acute rises in creatinine levels for most individuals with CKD. Furthermore, 2 weeks after treatment initiation may not be a sufficient time to assess renal function and recommendations for retesting to confirm a persistent rise in creatinine level before discontinuing RAASI therapy, as supported by our findings.^29^

### Strengths and Limitations

The strengths of the study lie largely within its natural outgrowth of patient care to address questions pertinent to a wide audience of health care professionals. Serum creatinine level was selected as the marker of kidney function, given its use in clinical practice, and a change of at least 30% was selected because this level has been advocated as a threshold for RAASI therapy discontinuation despite the limitations of creatinine level as an indicator of eGFR.^13,14,15^ Primary care practices were selected rather than specialty clinics to minimize the likelihood of off-site laboratory follow-up. Individuals receiving new diuretic prescriptions were included to contextualize these changes, given the prevalence of diuretic use in the outpatient setting in individuals with CKD and frequency of electrolyte disturbances requiring close laboratory monitoring. Care was taken to minimize comparisons between RAASI and diuretic prescriptions, given the risk of confounding by indication.

The study has several limitations. We required a follow-up laboratory measurement within 90 days of the medication prescription date because our goal was to investigate acute laboratory measurement changes. Laboratory values measured outside this period were less likely to be ordered for follow-up after a new RAASI or diuretic prescription. This time frame also gives individuals adequate opportunity to fill prescriptions and present for laboratory follow-up. Physicians may select closer laboratory follow-up for individuals with a higher burden of comorbidities or abnormal laboratories at baseline, thereby self-selecting for a higher risk population, and our data should be extrapolated cautiously. Similarly, we required 3 eGFR values within the year before the medication start date for our measure of CKD. As opposed to a single eGFR value, this criterion improves confidence in the diagnosis and stage of CKD, but as stated above, it may limit generalizability to individuals who may be selected for frequent monitoring.

Overall, the high frequency of laboratory monitoring in this cohort may be accounted for by a uniform health care network including primary care and specialty offices as well as affiliation with 2 academic teaching hospitals in a suburban or urban setting. There were no major differences in baseline comorbidities and laboratory values when the excluded participants were compared with the included participants. Nevertheless, the generalizability of the results may be limited owing to possible residual confounding by indication inherent in the inclusion criterion that required frequent laboratory testing. In addition, we were unable to evaluate between-practice variation or site-specific factors and thus were not able to control for center effects. However, differences in prescribing and laboratory monitoring practices between individual centers are likely to be mitigated by the very large numbers of prescribers, limited geographic region, uniform electronic medical record, and uniform academic affiliation of all health care professionals. However, we cannot rule out the possibility of differences in patient demographics, follow-up, and compliance between sites.

As in many pharmacoepidemologic studies, we were unable to capture medication adherence or indication for the new prescription. RAASIs and diuretics are prescribed with varying intent (eg, acute volume management, long-term blood pressure control), and we were unable to adjust for this in our analysis. However, our exposures were creatinine and potassium level disturbances on follow-up laboratory evaluations, which are less likely influenced by indication. The ED visits and hospitalizations may have been underreported because they were only captured within the health care network, although we would have expected hospitalization outside of our system to be nondifferential with respect to acute changes in levels of electrolytes and creatinine.

## Conclusions

We found that acute increase in serum creatinine levels of at least 30% and hyperkalemia were not associated with ED visits, hospitalizations, or mortality in participants who initiated RAASI therapy and that therapy discontinuation after an initial rise in creatinine levels also did not influence rates of sustained creatinine level increase, ED visits, hospitalizations, or mortality. Our data suggest that careful observation and serial monitoring may be a reasonable alternative to discontinuation in participants with modest changes in serum creatinine or potassium levels. Strategies of care focused on structured laboratory monitoring for sustained changes may maximize the benefits in long-term preservation of kidney function and cardiovascular protection over the acute risks of creatinine and potassium level changes.^2,11,14,25^ Additional studies to confirm our findings in diverse populations are warranted.

## Notes

Supplement 1. **eTable 1.** Incident Prescriptions of Renin Angiotensin Aldosterone System Inhibitors and Diuretics by Medication Name, 2009-2011 **eTable 2.** Comparison of Included and Excluded Participants by Drug Class **eTable 3.** Incidence of Creatinine Change in Individuals With an Initial Rise in Creatinine ≥30% From Baseline Categorized by Continuation or Discontinuation of New Renin Angiotensin Aldosterone System Inhibitor or Diuretic Prescription **eTable 4.** Incidence of Emergency Department Visit or Hospitalization and Mortality Within 1 Year of the First Follow-up Laboratory Measurement After Renin Angiotensin Aldosterone System Inhibitor or Diuretic Prescription **eTable 5.** Associations by Competing Risk Modeling With Emergency Department Visits or Hospitalizations Within 1 Year of the First Follow-Up Laboratory Measurment After Renin Angiotensin Aldosterone System Inhibitor Prescription With Death as a Competing Risk
