# Supplementary material for: Association of Changes in Creatinine and Potassium Levels After Initiation of Renin Angiotensin Aldosterone System Inhibitors With Emergency Department Visits, Hospitalizations, and Mortality in Individuals With Chronic Kidney Disease
Source: JAMA Netw Open. 2018 Nov 2;1(7):e183874. doi: 10.1001/jamanetworkopen.2018.3874 (PMC6324397; doi:10.1001/jamanetworkopen.2018.3874)
Supplement: Supplement 1. — eTable 1. Incident Prescriptions of Renin Angiotensin Aldosterone System Inhibitors and Diuretics by Medication Name, 2009-2011 eTable 2. Comparison of Included and Excluded Participants by Drug Class eTable 3. Incidence of Creatinine Change in Individuals With an Initial Rise in Creatinine ≥30% From Baseline Categorized by Continuation or Discontinuation of New Renin Angiotensin Aldosterone System Inhibitor or Diuretic Prescription eTable 4. Incidence of Emergency Department Visit or Hospitalization and Mortality Within 1 Year of the First Follow-up Laboratory Measurement After Renin Angiotensin Aldosterone System Inhibitor or Diuretic Prescription eTable 5. Associations by Competing Risk Modeling With Emergency Department Visits or Hospitalizations Within 1 Year of the First Follow-Up Laboratory Measurment After Renin Angiotensin Aldosterone System Inhibitor Prescription With Death as a Competing Risk [file jamanetwopen-1-e183874-s001.pdf]

Supplementary Online Content

Garlo KG, Bates DW, Seger DL, Fiskio JM, Charytan DM. Association of changes in creatinine and potassium levels after initiation of renin angiotensin aldosterone system inhibitors with emergency department visits, hospitalizations, and mortality in individuals with chronic kidney disease. *JAMA Netw Open.* 2018:1(6):e183874.
doi:10.1001/jamanetworkopen.2018.3874

eTable 1. Incident Prescriptions of Renin Angiotensin Aldosterone System Inhibitors and Diuretics by Medication Name, 2009-2011

eTable 2. Comparison of Included and Excluded Participants by Drug Class

eTable 3. Incidence of Creatinine Change in Individuals With an Initial Rise in Creatinine ≥30% From Baseline Categorized by Continuation or Discontinuation of New Renin Angiotensin Aldosterone System Inhibitor or Diuretic Prescription

eTable 4. Incidence of Emergency Department Visit or Hospitalization and Mortality Within 1 Year of the First Follow-up Laboratory Measurement After Renin Angiotensin Aldosterone System Inhibitor or Diuretic Prescription

eTable 5. Associations by Competing Risk Modeling With Emergency Department Visits or Hospitalizations Within 1 Year of the First Follow-Up Laboratory Measurment After Renin Angiotensin Aldosterone System Inhibitor Prescription With Death as a Competing Risk

This supplementary material has been provided by the authors to give readers additional information about their work.

© 2018 Garlo KG et al. *JAMA Network Open.*

eTable 1. Incident Prescriptions of Renin Angiotensin Aldosterone System Inhibitors and Diuretics by Medication Name, 2009-2011

**RAASi, N = 2,606, n (%)**	**Diuretic, N=2,568, n (%)**
Lisinopril 1,871 (71.8)	Furosemide 1,567 (61.0)
Valsartan 206 (7.9)	HCTZ 672 (26.2)
Losartan 159 (6.1)	Triamterene/HCTZ 152 (5.9)
Irbesartan 82 (3.1)	Torsemide 65 (2.5)
Spironolactone 86 (3.3), Eplerenone 3 (0.1)*	Chlorthalidone 30 (1.2)
Enalapril 55 (2.1)	Bumetanide 26 (1.0)
Combination RASi and diuretic 182 (3.4)*<br>Aldactazide (Spironolactone/HCTZ) 11 (6)<br>Benazepril/HCTZ 2 (1)<br>Candesartan/HCTZ 1 (0.5)<br>Enalapril/HCTZ 5 (3)<br>Irbesartan/HCTZ 8 (4)<br>Lisinopril/HCTZ 83 (46)<br>Losartan/HCTZ 21 (12)<br>Moexipril/HCTZ 2 (1)<br>Olmesartan/HCTZ 12 (6)<br>Valsartan/HCTZ 37 (20)	Combination beta blocker 17 (0.7)<br>Atenolol/Chlorthalidone 10 (0.4)<br>Bisoprolol/HCTZ 6 (0.2)<br>Metoprolol/HCTZ 1 (0.04)<br>Combination alpha blocker 3 (0.1)<br>Hydralazine/HCTZ 3 (0.1)

Data includes all incident prescription even if the medication was discontinued as well as combined renin angiotensin aldosterone system inhibitors/diuretics and mineralocorticoid receptor antagonists. All individuals had chronic kidney disease stage 3-5 (eGFR ≤60 ml/min/1.75m^2) not on dialysis and were newly prescribed a renin angiotensin aldosterone system inhibitor (RAASi) or diuretic during the study period 2009-2011. They also had baseline lab testing for renal function and one follow up lab within 90 days of the medication start date. *Mineralcorticoid receptor antagonists and aldosterone antagonists were excluded. RAASi = renin angiotensin aldosterone system inhibitor, HCTZ = hydrochlorothiazide.

© 2018 Garlo KG et al. *JAMA Network Open.*

eTable 2. Comparison of Included and Excluded Participants by Drug Class

	**RAASi (Excluded)** 1,521 (56.0%)	**RAASi (Included)** 2,354 (50.5%)	**Diuretic (Excluded** 1,194 (44.0%)	**Diuretic (Included)** 2,307 (49.5%)
Age (yrs), mean ±SD	72 ±12.9	70 ±13.5	75 ±12.5	73 ±13.8
Age ≥65 (%)	74.6	66.8	80.0	73.9
Sex, M (%)	55.6	49.3	65.8	43.1
CKD (%)				
Stage 3	94.6	92.3	91.5	57.7
Stage 4	4.0	6.5	6.9	10.0
Stage 5	1.4	1.2	1.6	1.2
Diabetes mellitus (%)	38.5	27.8	27.2	19.3
Hypertension (%)	83.6	61.0	83.3	56.1
Cardiovascular disease (%)	25.5	19.8	30.8	26.7
NSAIDs (%)	18.2	19.5	18.7	17.2
Baseline labs, mean ±SD				
Creatinine mg/dL	1.36 ±0.64	1.39 ± 0.6	1.37 ±0.59	1.48 ± 0.9
eGFR ml/min/1.732m^2	47.1 ±9.24	46.6 ± 10.2	45.6 ±10.27	44.7 ± 11.6
Potassium mmol/dL	4.4 ±0.46	4.3 ± 0.5	4.2 ±0.50	4.2 ± 0.5

© 2018 Garlo KG et al. *JAMA Network Open.*

eTable 3. Incidence of Creatinine Change in Individuals With an Initial Rise in Creatinine ≥30% From Baseline Categorized by Continuation or Discontinuation of New Renin Angiotensin Aldosterone System Inhibitor or Diuretic Prescription

**Change in creatinine from baseline on second follow up lab**					
	**Overall**<br>n = 249	**Med discontinued**<br>n = 226 (90.8%)	**Med continued**<br>n = 23 (9.2%)	**Univariate OR†**<br>**(95% CI)**	**p-value**
**Creatinine change < 30 %, n (%)**	146 (58.6)	136 (60.2)	10 (43.5)	1.96 (0.83-4.67)	0.13
Decreased	99 (39.8)	93 (41.2)	6 (26.1)	2.24 (0.82-6.15)	0.12
Unchanged, 0-30%	47 (18.9)	43 (19.0)	4 (17.4)	1.55 (0.48-5.04)	0.46
**Creatinine change ≥ 30 %, n (%)**	103 (41.4)	90 (39.8)	13 (56.5)	0.51 (0.21-1.21)	0.13
>30-50%	26 (10.4)	23 (10.2)	3 (13.0)	0.56 (0.14-2.2)	0.41
>50-200%	61 (24.5)	53 (23.5)	8 (34.8)	0.49 (0.20-1.24)	0.13
>200%	16 (6.4)	14 (6.2)	2 (8.7)		
**Change in creatinine from baseline on third follow up lab‡**					
	**Overall**<br>n = 41	**Med discontinued**<br>n = 9 (22.0%)	**Med continued**<br>n =32 (78.0%)		
**Creatinine change < 30 %, n (%)**	27 (65.9)	7 (77.8)	20 (62.5)		
Decreased	1 (2.4)	0 (0.0)	1 (3.1)		
Unchanged, 0-30%	26 (63.4)	7 (77.8)	19 (59.4)		
**Creatinine change ≥ 30 %, n (%)**	14 (34.1~~2~~)	2 (22.2)	12 (37.5)		
>30-50%	1 (2.4)	0 (0.0)	1 (3.1)		
>50-200%	3 (7.3)	1 (11.1)	2 (6.3)		
>200%	10 (24.4)	1 (11.1)	9 (28.1)		

†The odds ratio represents the proportion of with the designated creatinine changes on the second lab in participants who had an initial rise in creatinine ≥ 30 % on the first lab between medication discontinuation on the numerator and continuation on the denominator. The groups >50-200% and >200% were combined. ‡ Individuals with creatinine increase ≥30% from baseline on both first and second follow up labs. OR = odds ratio, CI = confidence interval. Of 1,353 participants with three follow up lab draws 41 had increases in creatinine ≥ 30 % on the first and second follow up labs.

© 2018 Garlo KG et al. *JAMA Network Open.*

eTable 4. Incidence of Emergency Department Visit or Hospitalization and Mortality Within 1 Year of the First Follow-up Laboratory Measurement After Renin Angiotensin Aldosterone System Inhibitor or Diuretic Prescription

	ED Visits & Hospitalizations No./Total No. (%)	Mortality No./Total No. (%)
Creatinine change[a]		
<30%	188/4,296 (4.4)	271/4,296 (6.3)
≥30%	22/365 (6.0)	42/365 (11.5)
Sustained creatinine change[b]		
<30%	15/146 (10.3)	20/146 (13.7)
≥30%	7/103 (6.8)	10/103 (9.7)
Potassium change[c]		
3.4-5.0 mEq/L	149/3444 (4.3)	226/3,444 (6.6)
>5.0 mEq/L	13/263 (4.9)	13/263 (4.9)
<3.4 mEq/L	8/196 (4.1)	27/196 (13.8)
Medication		
Diuretics	113/2,307 (4.9)	217/2,307(9.4)
RAASi	97/2,354 (4.2)	96/2,354(4.1)
Medication		
Continued	172/3830 (4.5)	248/3,830 (6.5)
Discontinued	38/831 (4.6)	45/831 (5.4)

[a]Initial change defined by the first follow up lab within 90 days of the prescription date. [b]Sustained creatinine change defined as creatinine ≥30 % of baseline on the first and second lab testing. The second follow up lab was within 365 days of prescription date. [c]All individuals had normal baseline serum potassium 3.5-5.0 mEq/L prior to medication start and a follow up potassium within 90 days of medication start.

© 2018 Garlo KG et al. *JAMA Network Open.*

eTable 5. Associations by Competing Risk Modeling With Emergency Department Visits or Hospitalizations Within 1 Year of the First Follow-Up Laboratory Measurment After Renin Angiotensin Aldosterone System Inhibitor Prescription With Death as a Competing Risk

	**SHR**	**95% CI**	**P Value**
Creatinine ≥30% of baseline[a]	1.02	0.45-2.32	.97
Sustained creatinine ≥30%[b]	1.02	0.63-1.62	.95
Hyperkalemia >5 mEq/L[c]	1.33	0.57- 3.11	.50
Medication continuation	1.34	0.67- 2.69	.41

[a]Initial change defined by the first follow up lab within 90 days of the prescription date. [b]Sustained creatinine change defined as creatinine ≥30 % of baseline on the first and second lab testing. The second follow up lab was within 365 days of prescription date. [c]All individuals had normal baseline serum potassium 3.5-5.0 mEq/L prior to medication start and a follow up potassium within 90 days of medication start. SHR = subdistribution hazard ratio, CI = confidence interval.

© 2018 Garlo KG et al. *JAMA Network Open.*
